# Changing Trends in the Clinical Presentation and Incidence of Molar Pregnancy in Saudi Arabia: A 30-Year Retrospective Analysis

**DOI:** 10.7759/cureus.50936

**Published:** 2023-12-22

**Authors:** Ayman Altalib, Noura Al Qahtani, Shrouq S Alosaimi, Mariam S Al Hashem, Roaa Almowallad, Maryam Al-Rufiei, Lujain I Alhumaid

**Affiliations:** 1 Obstetrics and Gynaecology, Imam Abdulrahman Bin Faisal University, Khobar, SAU

**Keywords:** hydatidiform mole, molar pregnancy, incidence, clinical presentation, changes

## Abstract

Background

Molar pregnancy (MP) incidence and clinical presentation vary significantly worldwide. Recent trends show changes in its clinical representation and incidence, particularly with the adoption of early diagnosis using first-trimester ultrasonography, which has reduced the prevalence of classical second-trimester presentations. This study aimed to analyze the changes in clinical presentation and incidence of MP among the Saudi population over the past 30 years.

Methods

In this retrospective study at King Fahad University Hospital, 121 complete mole (CM) pregnancy cases diagnosed and pathologically confirmed were reviewed. This included 87 cases from 2007 to 2022 (recent group) and 34 cases from 1992 to 2006 (older group). Cases of CM diagnosed before January 1992 and other diagnoses such as PM, invasive mole, or choriocarcinoma were excluded; thus, this study is focused on CM in particular. We compared patient age, gravidity, parity, abortion history, gestational age at diagnosis, hyperemesis gravidarum symptoms, anemia symptoms, and hemoglobin levels. Classical symptoms and signs related to CM were also reviewed. Data were analyzed using Microsoft Excel 2021 (Microsoft Corporation, Redmond, Washington, United States) and presented as mean, frequency, and percentage, with chi-squared tests for categorical variables; p<0.05 was considered statistically significant.

Results

The incidence of CM declined from 2.1 per 1,000 deliveries to 0.9 per 1,000 deliveries. Vaginal bleeding was the most common presentation in both the older (91.9%) and recent (67.6%) groups. Hyperemesis gravidarum prevalence was similar in both groups. Theca-lutein cysts were more frequent in the older group (27.5%) than the recent group (8.8%). A significant difference was observed in the occurrence of a large-for-date uterus between the older (63.20%) and recent (23.5%) groups. Notably, 14.7% of patients in the recent group were asymptomatic at diagnosis. Anemia was present in 46 cases (52.8%) of the older group but absent in the recent group, and preeclampsia occurred in 10 cases (11.4%) of the older group but not in the recent group.

Conclusions

Advancements in ultrasound technology, including transvaginal probes with Doppler capabilities, have enabled earlier pregnancy diagnosis, as early as five to six weeks of gestation. Many MP are now diagnosed in the first trimester without the classic clinical symptoms or "snow-storm" ultrasound appearance. The availability of sensitive beta-human chorionic gonadotropin assays has led to the early termination of these pregnancies, marking a significant shift in the management of MP.

## Introduction

Hydatidiform mole (HM), a benign gestational trophoblastic neoplasm, presents either as a complete mole (CM) or a partial mole (PM). These forms differ in their histopathological and genetic characteristics. CM is the more prevalent, accounting for 90% of HMs. HM's incidence varies geographically; for instance, in the Republic of Korea, the incidence is 1.1 per 1,000 pregnancies [1], while in Stockholm, it is 2.08 per 1,000 pregnancies [2]. In Italy and Turkey, the incidences are 2.3 and 0.7 per 1,000 pregnancies, respectively [3,4]. In Saudi Arabia, the incidence decreased from one in 446 pregnancies in 1988 to 0.9 per 1,000 in 2016 [5,6], possibly due to improvements in diet and socioeconomic conditions [7].

Risk factors for HM include extreme maternal age and a history of HM. Over recent decades, its clinical presentation has evolved. Historically, symptoms like excessive uterine enlargement, ovarian enlargement from theca-lutein cysts, preeclampsia, hyperemesis gravidarum, thyrotoxicosis, and anemia with elevated beta-human chorionic gonadotropin (ß-hCG) were common in the second trimester but are now rare.

Diagnosis of molar pregnancy (MP), traditionally identified in the late first or early second trimester by a "snow-storm" ultrasound appearance, is now more commonly made in the first trimester. This shift is due to the typical appearance of a complex, echogenic intrauterine mass with small cystic spaces. Histological examination of the conception products is crucial; without it, HM can be missed. Earlier detection has changed the pathology, showing less trophoblastic hyperplasia and hydropic villi [8].

Several studies indicate a shift in the clinical presentation of HM, with classical symptoms becoming increasingly rare [9-11]. This trend is linked to the widespread use of ultrasonography and early referral for issues like pregnancy dating or vaginal bleeding, facilitating earlier HM diagnosis and termination before complications arise. Notably, this earlier detection has not altered the time to remission or progression to gestational trophoblastic neoplasia [12]. This study aims to investigate the changes in clinical presentation and incidence of HM in Saudi Arabia over the past 30 years. By examining these alterations, this research hopes to alert medical professionals to the fact that HM can manifest with a variety of symptoms, not only the severe traditional ones. This could ultimately result in an earlier diagnosis and better treatment outcomes for HM-affected women.

## Materials and methods

Study design and population

This retrospective study examined cases of CM pregnancy diagnosed at King Fahad University Hospital, a tertiary referral hospital in Eastern Saudi Arabia. The study period was divided into two cohorts: the "recent group" (January 2007 to September 2022) and the "older group" (1992 to December 2006). These periods were allocated to be 15 years for each group because it's the maximum period that could provide us with adequate data regarding the older group, nevertheless, to keep both groups equal in period as possible. Cases of CM diagnosed before January 1992 and other diagnoses such as PM, invasive mole, or choriocarcinoma were excluded. Moreover, some data were not statically analyzed and ultimately not used in this study due to its incomplete availability. The study received ethical approval from the Institutional Review Board of Imam Abdulrahman Bin Faisal University (approval number: IRB-UGS-2022-01-461).

Data collection

Data were collected from the hospital's ward logbook and health information system. Recorded information included patient demographics (age, gravidity, parity, abortion history), clinical details (gestational age at diagnosis, symptoms of hyperemesis gravidarum, anemia), and diagnostic findings (hemoglobin level, classical CM signs like preeclampsia and hyperthyroidism, ultrasonographic findings, and ovarian enlargement by theca-lutein cysts).

Diagnostic criteria and measurements

Ultrasonography was performed differently in the two groups: transabdominal in the older group and transvaginal in the recent group. Uterine volume, considered excessive if at least three to four weeks greater than the expected gestational age, was also assessed. Hyperemesis gravidarum was defined as vomiting more than three times daily with weight loss exceeding 3 kg or 5% of body weight and ketonuria [13]. Early-onset preeclampsia and hyperthyroidism were defined using specific clinical criteria, including blood pressure and symptomatology. Anemia was classified as a hemoglobin level below 11 g/dL in the first trimester and 10.5 g/dL in the second trimester [14].

Variables and data analysis

Independent variables comprised patient age, parity, and the time period (i.e., older and recent groups). Dependent variables included various clinical presentation symptoms. The study controlled for the diagnosis of CM. Data were entered and analyzed using Microsoft Excel 2021 (Microsoft Corporation, Redmond, Washington, United States). The results were presented as mean±standard deviation, frequency, percentage, and distribution for categorized variables.

## Results

From 1992 to 2006, there were 40,700 deliveries at our facility, with 87 cases of CM pregnancy, equating to 2.1 per 1,000 deliveries. In contrast, from 2007 to 2022, out of 37,500 deliveries, there were 34 cases of CM or 0.9 cases per 1,000 deliveries. The mean gestational age at diagnosis in the older group (1992-2006) was 14 weeks, five days, while in the recent group (2007-2022), it was 10 weeks, three days.

Table 1 presents the frequency distribution of age and parity among patients. In the recent group, patient ages ranged from 18 to 36 years, with a mean age of 32.2 years. In the older group, ages spanned from 16 to 45 years, with a mean of 28.9 years. Patients were categorized into five age intervals: 20 years or younger, 21-25 years, 26-30 years, 31-35 years, and 36 years or older. In the recent group, CM cases were more frequent in patients under 20 years old (23.5%) compared to the older group (5.74%), with a significant difference (p=0.05). In the older group, CM was more prevalent among women aged 31 to over 36 years (26.4%), while in the recent group, it was significantly higher in women aged 36 years or older (52.9%; p=0.01).

**Table 1:** Frequency distribution of age and parity for patients with molar pregnancy NS: statistically nonsignificant

Variable	Older group, n=87 (%)	Recent group, n=34 (%)	p-value
Age (years)			
≤20	5 (5.74%)	8 (23.5%)	0.005
21-25	16 (18.3%)	4 (11.7%)	0.38 NS
26-30	20 (22.98%)	2 (5.88%)	0.03
31-35	23 (26.4%)	2 (5.88%)	0.01
≥36	23 (26.4%)	18 (52.9%)	0.01
Parity			
P0	6 (6.9%)	15 (44.1%)	<0.001
P1	9 (10.3%)	5 (14.7%)	0.50 NS
P2	16 (18.3%)	4 (11.7%)	0.54 NS
P3	20 (22.9%)	4 (11.7%)	0.26 NS
P4	36 (41.3%)	6 (17.6%)	0.01

Parity in patients ranged from 0 to over 4. In the older group, CM was more common in multiparous women (41.3%) than nulliparous women (6.9%; p=0.01). Conversely, 44.1% of CM cases in the recent group were in nulliparous women compared to 17.6% in multiparous women, indicating a significant difference (p=0.001).

Table 2 outlines the clinical presentation of patients in both groups. Vaginal bleeding was the most common symptom in both the older and recent groups (91.9% vs. 67.6%, respectively, p<0.001). The incidence of hyperemesis gravidarum was similar in both groups (29.8% in the older group vs. 29.4% in the recent group, p=0.96). Thyrotoxicosis occurred in 3.4% of cases in the older group and 2.9% in the recent group (p=0.89). Ovarian enlargement due to theca-lutein cysts was more frequently observed in the older group (27.5%) compared to the recent group (8.8%, p=0.02).

**Table 2:** Clinical presentation of patients with molar pregnancy NS: statistically nonsignificant

Signs and symptoms	Older group, n=87 (%)	Recent group, n=34 (%)	p-value
Vaginal bleeding	80 (91.9%)	23 (67.6%)	<0.001
Hyperemesis gravidarum	26 (29.8%)	10 (29.4%)	0.96 NS
Hyperthyroidism/thyrotoxicosis	3 (3.4%)	1 (2.9%)	0.89 NS
Large-for-date uterus	55 (63.2%)	8 (23.5%)	<0.001
Small-for-date uterus	12 (13.7%)	6 (17.6%)	0.59 NS
Equal-for-date uterus	23 (26.4%)	12 (35.3%)	0.33 NS
Ovarian cyst	24 (27.5%)	3 (8.8%)	0.02
Asymptomatic	0	5 (14.7%)	0.001
Anemia	46 (52.8%)	0	<0.0001
Preeclampsia	10 (11.4%)	0	0.001

A notable finding was the significant difference in cases presenting with a large-for-date uterus between the two groups: 63.20% in the older group and 23.5% in the recent group (p<0.001). However, the incidence of small-for-date and equal-for-date uterus presentations was similar between the groups. Interestingly, 14.7% of patients in the recent group were diagnosed early in the first trimester while asymptomatic (p=0.001), a scenario not observed in the older group. Anemia was present in 52.8% of the older group cases but absent in the recent group (p=0.001). Similarly, preeclampsia was noted in 11.4% of the older group but not in the recent group (p=0.001).

## Discussion

The incidence of MP varies globally due to differences in methodologies such as data reporting (population-based or hospital-based), mole classifications, and case detection. Recently, there has been a global decline in MP incidence. In 1988, two local studies from Saudi Arabia reported incidences of one in 446 and one in 676 pregnancies, respectively [15,16]. A more recent 2016 report from Saudi Arabia indicated an incidence of 0.9 per 1,000 pregnancies [17]. Similar declining trends have been observed in other countries, such as South Korea, where the incidence dropped from 4.4 per 1,000 pregnancies in the 1960s to 1.6 in the 1990s [18], and in Eastern Nepal, from 4.17 to 2.58 per 1,000 pregnancies [19]. Risk factors for MP include extreme maternal age, previous MP, ethnicity, geographic area, dietary factors, and blood groups. Animal studies suggest that diet can influence genetic predispositions [7] and nutritional deficiencies, particularly in vitamin A and folates, are linked to the development of CM pregnancy, leading to the production of immature ovum [20].

Since the 1995 report by Soto-Wright et al. [21], many studies have confirmed changes in the clinical presentation of MP [8-10,19,22]. The widespread use of transvaginal ultrasound in the first trimester has enabled the earlier diagnosis of HM before the appearance of complications and classical "snow-storm" ultrasound images and less marked histopathological features than traditionally seen.

Extreme maternal age is a well-recognized risk factor for CM development. Adolescents face a sevenfold higher risk compared to average-aged women, while women over 40 have nearly double the risk [23]. Our study aligns with Mangili et al. [9] in that most cases were in women aged 36 and older, but we also observed a significant increase in cases among women aged 20 years and younger, potentially due to early marriages in rural areas served by our center. The recent group showed a significant rise in nulliparous women, though the impact of nulliparity on CM development remains unclear. Notably, most CM in the older group was diagnosed in the second trimester at a mean gestational age of 14+5 weeks, compared to the first trimester in the recent group at 10+3 weeks, as reported in several studies [8,10,21,22].

Vaginal bleeding, traditionally the most common presenting symptom, was seen in 92% of the older group compared to 67% in the recent group. This finding is consistent with Soto-Wright et al. [21] and Mangili et al. [9], who reported a decrease in vaginal bleeding as a presenting symptom over time. Large-for-date uterus presentations were more prevalent in the older group (63.2%) and less in the recent group (24%). While Braga et al. [10] found a downward trend in large-for-date uterus presentations, it was not statistically significant (p=0.65). Theca-lutein cysts were more common in the older group (28%) compared to the recent group (9%). Mangili et al. [9] and Braga et al. [10] reported varying trends in the presence of theca-lutein cysts over time.

Using high-resolution transvaginal ultrasound and sensitive, quantitative ß-hCG assays has enabled earlier asymptomatic diagnosis in 15% of recent group cases [9,11]. Anemia, secondary to prolonged bleeding, was prevalent in the older group (52.8%) but absent in the recent group, aligning with findings by Mangili et al. [9] and Agrawal et al. [19], who also reported high rates of anemia and blood transfusions. Preeclampsia, found in 11.4% of the older group, was absent in the recent group [8,9,17].

Our center's policy of submitting any product of conception for histopathological examination and counseling patients post abortion or pregnancy termination about contraception has been crucial. This is especially important as early CM diagnosis might not present with classical histologic features, necessitating careful evaluation by histopathologists.

This study represents the first to examine changes in MP presentation in Saudi Arabia in over 30 years. Data from 1992 to 2022 were accurately obtained from patients' medical records. However, limitations include its retrospective design, reliance on data from a single tertiary referral hospital, the small sample size of MP cases, recall bias, selection bias, information bias, and reverse causality bias which all may impact the generalizability and acuity of results.

## Conclusions

Over the past decade, advancements in ultrasound technology, particularly transvaginal probes with Doppler capabilities, and the use of sensitive ß-hCG assays have enabled the early diagnosis of MP, often as early as five to six weeks of gestation and frequently without the classic clinical symptoms or "snow-storm" ultrasound appearance. This early detection, coupled with increased patient awareness, early antenatal clinic visits, and first-trimester ultrasound referrals, has contributed to a decline in the incidence and altered the clinical presentation of MP in Saudi Arabia. Our study reveals these shifts, emphasizing the importance of understanding the evolving patterns of MP for improved diagnosis, management, and patient counseling, particularly for women with higher risk profiles, to enhance outcomes and reduce complications. This research offers valuable insights into the changing landscape of MP in Saudi Arabia, with practical implications for enhancing patient care. Subsequent research directions could examine the alterations in clinical presentation from an alternative standpoint, thereby identifying additional plausible reasons for this noted shift.
